# Spontaneous Cerebrospinal Fluid Rhinorrhea Secondary to Idiopathic Intracranial Hypertension

**DOI:** 10.7759/cureus.42353

**Published:** 2023-07-24

**Authors:** Mohamed Ali, Moayad A Elgassim, Hamad M Faisal, Amin Saied Sanosi Saied, Mohamed Elgassim

**Affiliations:** 1 Internal Medicine, Hamad Medical Corporation, Doha, QAT; 2 Medical School, Taylor's University Lakeside Campus, Subang Jaya, MYS

**Keywords:** intracranial hypertension, idiopathic, rhinorrhea, cerebrospinal fluid, spontaneous

## Abstract

The cerebrospinal fluid (CSF) is a physiological fluid that functions to protect the brain tissue and maintain intracranial pressure. Defects between the subarachnoid spaces and other spaces can cause CSF leaks. We report the case of a 37-year-old female with no known past medical history who presented to the emergency department with a history of headaches for two months, nasal drip for 1.5 months, and recurrent fevers. Idiopathic intracranial hypertension was confirmed by cranial magnetic resonance imaging (MRI) and transnasal endoscopic repair of a CSF leak defect, and an abdomen fat graft was performed followed by an Axium navigation-guided right ventriculoperitoneal shunt (VPS).

## Introduction

The cerebrospinal fluid (CSF) is the physiological fluid produced by the choroid plexus. It functions to protect both the brain tissue and the intracranial pressure (ICP). A CSF leak may occur when there is a defect between the subarachnoid spaces and other spaces, usually resulting from meningeal damage. The most common cause of CSF leaks is head injuries, which account for more than four out of five cases [1]. Cerebrospinal fluid leaks are primarily due to structural distortion as a result of craniofacial trauma, which represents 80% of the leaks. In comparison, 16% are a consequence of iatrogenic causes, and the last 4% are attributed to spontaneous leak defects and congenital defects [2]. The outcome of CSF leaks depends on early diagnosis and management [3]. However, spontaneous CSF leaks are uncommon, hence the late detection and management [4]. Current treatments include intravenous acetazolamide and repair in the case of an apparent defect [3].

## Case presentation

**Figure 1:**
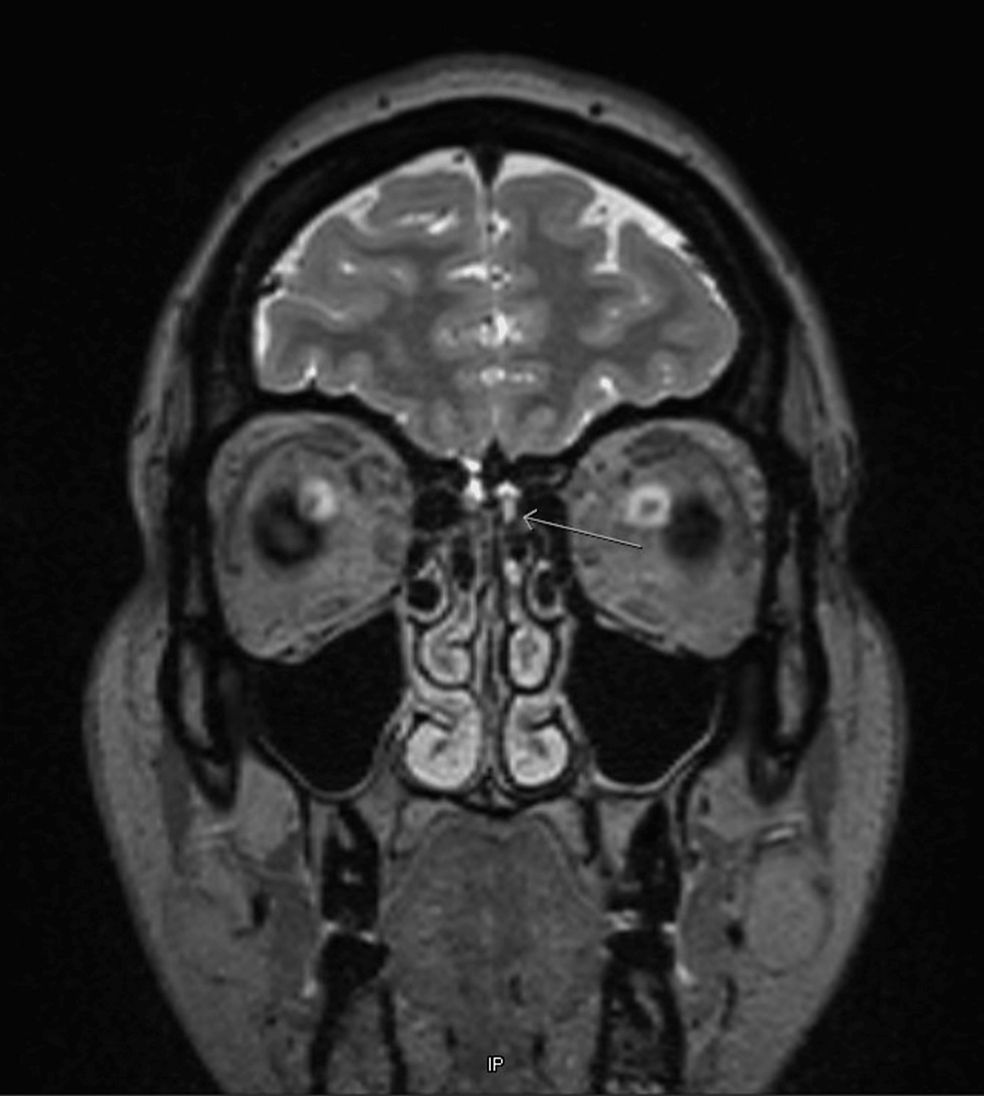
The head CT scan shows a widened empty sella and slightly tortuous optic nerves with a prominent perioptic CSF space.

A 37-year-old female with no known previous medical illness or surgeries presented to the emergency department with a two-month history of headaches, a 1.5-month history of nasal drip, and recurrent fevers. The headache was bifrontal, pressing in quality, highest in the morning, increased when she bent forward, and was of moderate severity. Nasal discharge was almost continuous, transparent, water-like, and left a salty taste if she swallowed it. She also had recurrent subjective fevers during this period, but they were never documented. Valsalva maneuvers were not tested to examine any association between the CSF leak and increased abdominal pressure. Initial assessment in the emergency department revealed that the patient was generally well, had normal vitals, and was afebrile. Examination by the Dandy maneuver showed a positive, clear fluid leak from the left nostril upon leaning forward. Examination of the nostril showed no sign of inflammation or blockage and was unable to pinpoint the area of leakage. Endoscopic examination showed clear discharge in the nose, mostly in the left sphenoethmoid recess, and minimal septal deviation to the left side. Fundoscopy performed showed grade 1 mild papilledema indicative of increased intracranial pressure. An initial computerized tomography (CT) scan of the head showed widened empty sella, and slightly tortuous optic nerves with a prominent perioptic CSF space that can represent benign intracranial hypertension (Figure 1).

**Figure 2:**
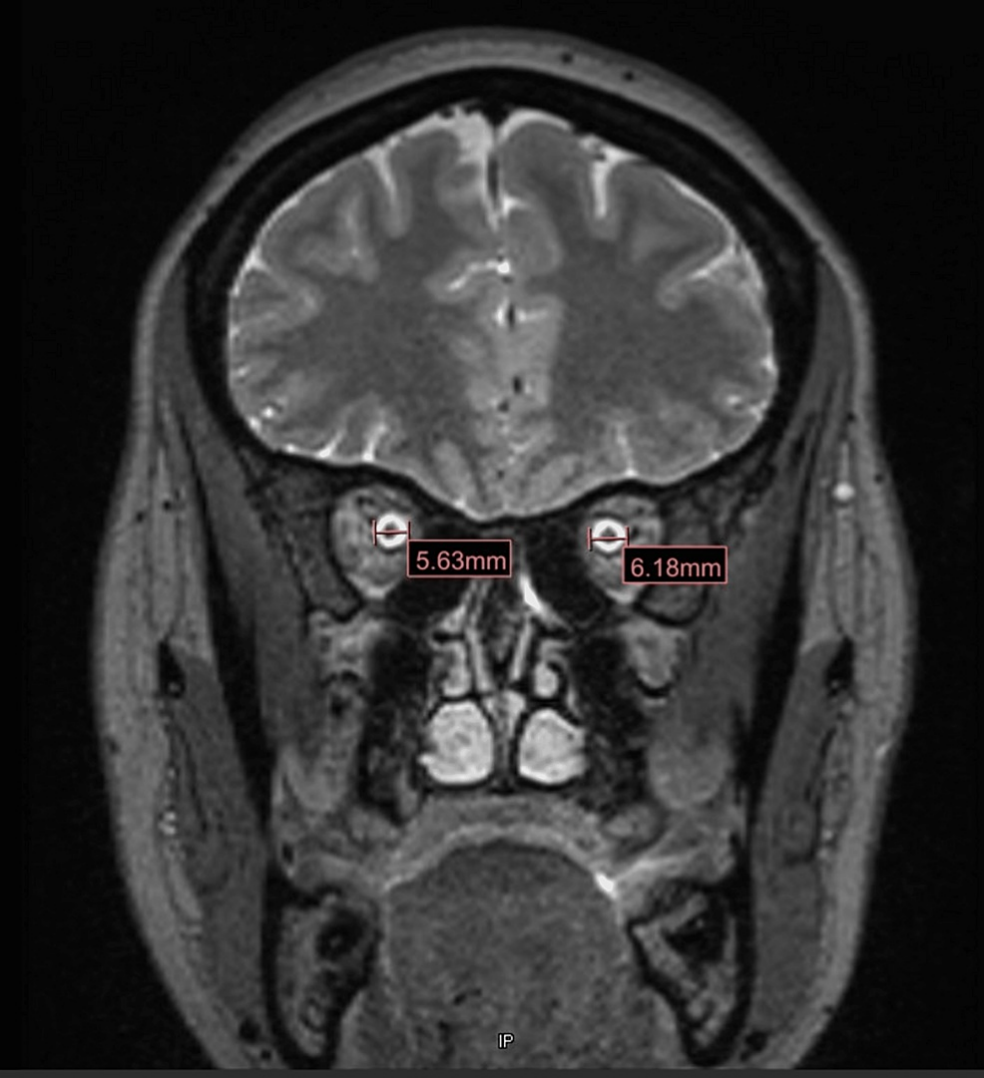
The repeat head CT scan reveals minimal mucosal lining thickening at the left side of the sphenoid sinus, bilateral middle concha bullosa, and patent ostiomeatal complexes.

A repeat CT scan revealed minimal mucosal lining thickening at the left side of the sphenoid sinus, otherwise clear paranasal sinuses, bilateral middle concha bullosa, and patent ostiomeatal complexes (Figure 2).

**Figure 3:**
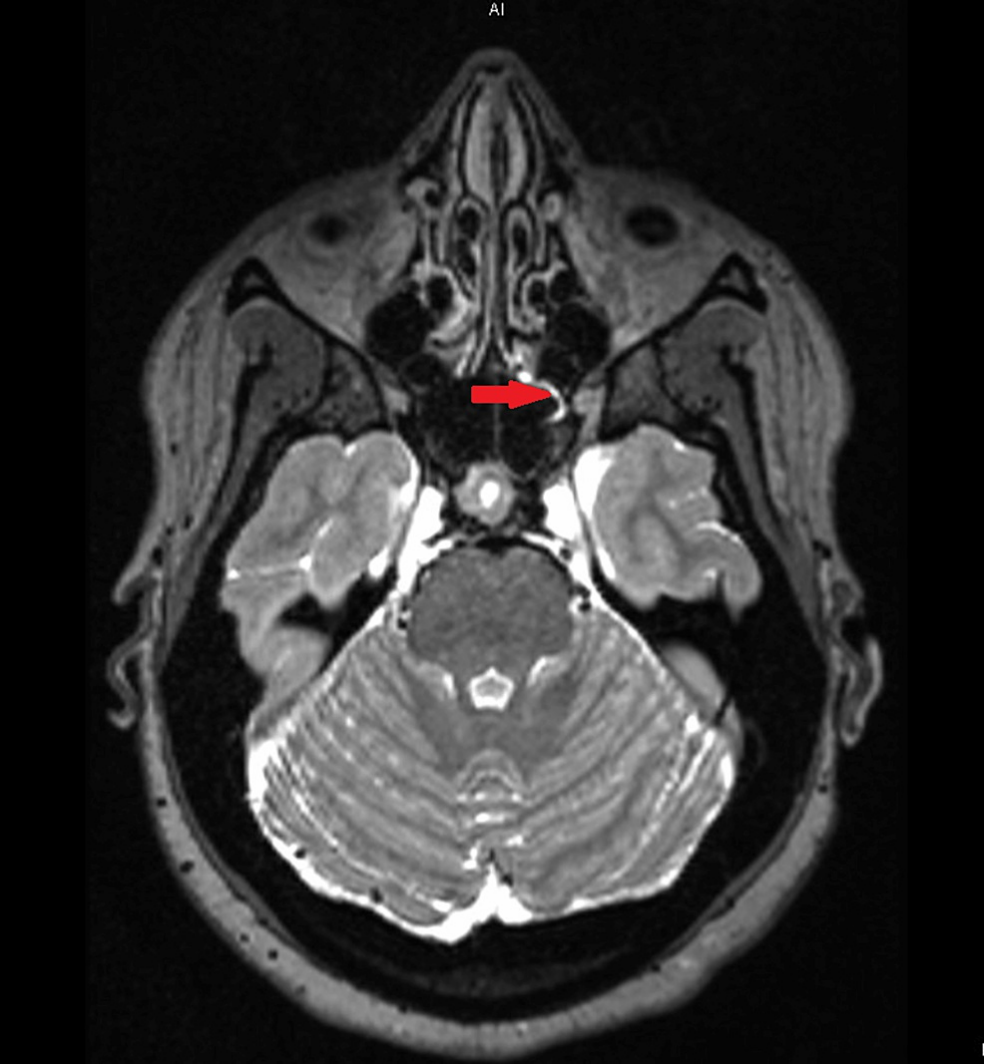
The cranial MRI confirms idiopathic intracranial hypertension and shows that the left olfactory gyrus is mildly sagging or protruding into the olfactory groove with scattered CSF signal intensity in the left ethmoid air cell, along the left sphenoethmoidal recess, and dependent in the left maxillary sinus.

Cranial magnetic resonance imaging (MRI) with contrast was done, which suggested idiopathic intracranial hypertension and showed that the left olfactory gyrus was mildly sagging or protruding into the olfactory groove with scattered CSF signal intensity in the left ethmoid air cell, along the left sphenoethmoidal recess, and dependent in the left maxillary sinus (Figure 3).

The superior sagittal sinus was ruled out. There was a suspected tiny fistulous tract from the left olfactory groove into the ethmoid sinus, as seen in Figure 1. All the aforementioned findings suggested a small left olfactory groove cephalocele with a CSF leak.

The patient refused to do a lumbar puncture; hence, CSF was not sent for testing. Fundoscopy performed showed grade 1 mild papilledema indicative of increased intracranial pressure. The neurosurgical team, with the support of the Ear, Nose, and Throat (ENT) team, performed a transnasal endoscopic repair of the CSF leak defect and abdomen fat graft, followed by an Axium navigation-guided right ventriculoperitoneal shunt (VPS) using a Medtronic programmable strata 2 with a valve pressure of 1.5. The patient was kept in the ward for four days after the operation for observation. She was seen 11 days postoperatively in the neurosurgery clinic when the rhinorrhea had stopped, but she was still having minor mucus discharge from both nostrils and continuing to have bifrontal headaches. She was seen once more 25 days after the operation in the ENT clinic, where she showed resolution of all symptoms with only minor crusting in the nasal cavity.

Electrophoresis and immunofixation showed the presence of beta-2 (B2) transferrin. This is indicative of the presence of CSF in the fluid.

## Discussion

A defect in the skull base can result in cerebrospinal fluid (CSF) leakage as it connects the nasal cavity with the subarachnoid space. Only 5% of reported cases of CSF leakage are associated with non-traumatic causes [5]. An increase in body mass index (BMI) >30 [6], female [7], and elevated intracranial pressure have been found to be the main associated factors with a non-traumatic CSF leak [6]. When the fundoscopy was performed, it showed grade 1 mild papilledema indicative of increased intracranial pressure. Nontraumatic CSF leaks are commonly due to idiopathic intracranial hypertension [3], which our patient had evidence of on clinical examination and radiological imaging.

Previous studies showed that persistently high intracranial pressure can lead to skull base defects leading to CSF leaks [8]. Increased weight causes low venous return due to raised intra-abdominal, cardiac, and pleural pressures. The lessened venous return from the brain leads to intracranial hypertension [8].

The first symptoms to present in spontaneous CSF leak and idiopathic intracranial hypertension are pulsating tinnitus and headache [9]. Our patient had the latter for two months, followed by a nasal drip. While the main presenting feature of idiopathic intracranial hypertension is the existence of unilateral or bilateral papilledema upon clinical examination, which was present in our patient, the papilledema may be mild or severe based on intracranial pressure (ICP) [10].

The investigation of choice to differentiate rhinorrhea due to CSF leak from other rhinorrhea causes is beta-2 (B2) transferrin, which carries a specificity of 95% and a sensitivity of 100%. Our patient had positive B2 transferrin, and the physician's clinical judgment as well as radiological findings confirmed the diagnosis [11,12].

In contrast, the most accurate methods of differentiating non-spontaneous rhinorrhea from spontaneous CSF rhinorrhea are computerized tomography (CT) scans and high-resolution magnetic resonance imaging (MRI). Although these two methods do not demonstrate the leak, they help in the localization of leaks when the leak is related to traumatic fractures or masses.

The gold standard for determining the amount as well as the origin of the CSF leak is the use of magnetic resonance/computerized tomography (MR/CT) cisternography. The downside to this technique is its redundant invasiveness, which is not required to establish a diagnosis of CSF leak [8]. Imaging in our patient showed a suspected tiny fistulous tract from the left olfactory groove into the ethmoid sinus.

In non-operative management of spontaneous CSF leaks, acetazolamide can be given; alternatively, we can pursue surgical options in case of failure. [13] Surgical interventions can be classified into extracranial and intracranial interventions. Intracranial intervention carries a significant probability of morbidity as well as a failure chance of over one-fifth. In contrast, extracranial intervention has a high chance of success in nine out of 10 cases and a low risk of morbidity [3].

Intracranial pressure tends to increase after surgical closure of the leakage due to diminished CSF drainage [14]. CSF shunts such as ventriculoperitoneal shunts can decrease the leak recurrence rate after surgical repair [15], hence dual management using a ventriculoperitoneal shunt and endoscopic repair is likely to be helpful [16].

Our neurosurgical team, with the support of the ENT team, performed a transnasal endoscopic repair of the CSF leak defect, followed by an Axium navigation-guided right VP shunt. This was done to avoid the recurrence of spontaneous CSF leakage after the repair of the defect.

## Conclusions

Spontaneous cerebrospinal fluid (CSF) rhinorrhea is an uncommon finding. It is associated with a high body mass index (BMI), increased intracranial pressure (ICH), and female gender. It commonly presents with headaches and tinnitus. Magnetic resonance/computerized tomography (MR/CT) cisternography is the gold standard for detecting the tract of the CSF leak. Management can be conservative (acetazolamide) or surgical (right ventriculoperitoneal shunt).
